# CETP and SGLT2 inhibitor combination therapy increases glycemic control: a 2x2 factorial Mendelian randomization analysis

**DOI:** 10.3389/fendo.2024.1359780

**Published:** 2024-06-19

**Authors:** Bohdan B. Khomtchouk, Patrick Sun, Zane A. Maggio, Marc Ditmarsch, John J. P. Kastelein, Michael H. Davidson

**Affiliations:** ^1^ Department of BioHealth Informatics, Luddy School of Informatics, Computing, and Engineering, Indiana University, Indianapolis, IN, United States; ^2^ Krannert Cardiovascular Research Center, Indiana University School of Medicine, Indianapolis, IN, United States; ^3^ Center for Diabetes and Metabolic Diseases, Indiana University School of Medicine, Indianapolis, IN, United States; ^4^ Center for Computational Biology and Bioinformatics, Indiana University School of Medicine, Indianapolis, IN, United States; ^5^ Department of Medical and Molecular Genetics, Indiana University School of Medicine, Indianapolis, IN, United States; ^6^ The College of the University of Chicago, Chicago, IL, United States; ^7^ NewAmsterdam Pharma B.V., Naarden, Netherlands; ^8^ Department of Vascular Medicine, Academic Medical Center, University of Amsterdam, Amsterdam, Netherlands; ^9^ Department of Medicine, Section of Cardiology, University of Chicago, Chicago, IL, United States

**Keywords:** Mendelian randomization, CETP, SGLT2, glycemic control, diabetes, epidemiology, combination therapy, cardioinformatics

## Abstract

**Introduction:**

Cholesteryl ester transfer protein (CETP) inhibitors, initially developed for treating hyperlipidemia, have shown promise in reducing the risk of new-onset diabetes during clinical trials. This positions CETP inhibitors as potential candidates for repurposing in metabolic disease treatment. Given their oral administration, they could complement existing oral medications like sodium-glucose cotransporter 2 (SGLT2) inhibitors, potentially delaying the need for injectable therapies such as insulin.

**Methods:**

We conducted a 2x2 factorial Mendelian Randomization analysis involving 233,765 participants from the UK Biobank. This study aimed to evaluate whether simultaneous genetic inhibition of CETP and SGLT2 enhances glycemic control compared to inhibiting each separately.

**Results:**

Our findings indicate that dual genetic inhibition of CETP and SGLT2 significantly reduces glycated hemoglobin levels compared to controls and single-agent inhibition. Additionally, the combined inhibition is linked to a lower incidence of diabetes compared to both the control group and SGLT2 inhibition alone.

**Discussion:**

These results suggest that combining CETP and SGLT2 inhibitor therapies may offer superior glycemic control over SGLT2 inhibitors alone. Future clinical trials should investigate the potential of repurposing CETP inhibitors for metabolic disease treatment, providing an oral therapeutic option that could benefit high-risk patients before they require injectable therapies like insulin or glucagon-like peptide-1 (GLP-1) receptor agonists.

## 1 Introduction

Metabolic disease has some of the highest medical burden in the world, with nearly 35% of people (over 100 million patients) afflicted with some form of metabolic syndrome in the United States alone, costing the healthcare system >$250 billion annually (1). Despite the extensive amount of research performed on developing treatments for metabolic disease, there is an unmet need for understanding synergies between existing treatment when combined in combination therapies, which has been a major driving force behind improved outcomes in other disease areas such as oncology. Furthermore, the growing understanding of an intersection between cardiovascular and metabolic disease represents a rich knowledge-base from which combination therapies using treatments from these two intersecting disease verticals can be used to develop innovative therapies to achieve better outcomes. Here, we investigate whether combining the hyperlipidemia drug class of cholesteryl ester transfer protein (CETP) inhibitors with the existing hyperglycemia treatment in sodium glucose transporter 2 (SGLT2) inhibitors is an effective strategy to improve glycemic control. SGLT2 inhibitors are a novel class of oral antidiabetic drugs that reduce glucose toxicity by stimulating its excretion into urine and inhibiting its reabsorption in the kidneys (2, 3). Because this mechanism of action is independent of insulin secretion or action, SGLT2i can be used in combination with other therapies to improve outcomes for patients afflicted with type II diabetes mellitus (T2DM) (4). Circulating levels of plasma CETP reduce pancreatic β-cell insulin secretion by disrupting cholesterol homeostasis through accumulation of free cholesterol, which causes β-cell lipotoxicity and dysfunction that induces T2DM (5). Therefore, CETP inhibition is one promising approach to ameliorating β-cell function in T2DM by decreasing islet cholesterol accumulation and inflammation (5). Since CETP inhibitors (CETPi) are known to increase HDL concentrations, which are the predominant acceptors of cell cholesterol and have been reported to inhibit β-cell apoptosis and promote β-cell survival, it follows that this therapeutic strategy may be important for maintaining normal β-cell function and insulin secretion (6). Indeed, previous meta-analyses of existing randomized controlled clinical trials of CETPi therapies have shown that CETP inhibitors significantly reduce the incidence of new onset diabetes while improving glucose homeostasis and metabolism (7). Therefore, in this study, we speculated that SGLT2i and CETPi combination therapy may be a promising strategy to treat type 2 diabetes. We computationally tested this hypothesis through the design of a new 2x2 factorial Mendelian randomization study comprised of 233,765 individuals from the UK Biobank. Our study builds upon and improves on prior work exploring the efficacy of combination therapies in smaller populations that focused on other clinical indications and drugs [e.g., genetically mimicking the effects of ezetimibe and statins in 108,376 people with coronary heart disease (8)].

## 2 Materials and methods

### 2.1 Construction of genetic scores

**Figure 1:**
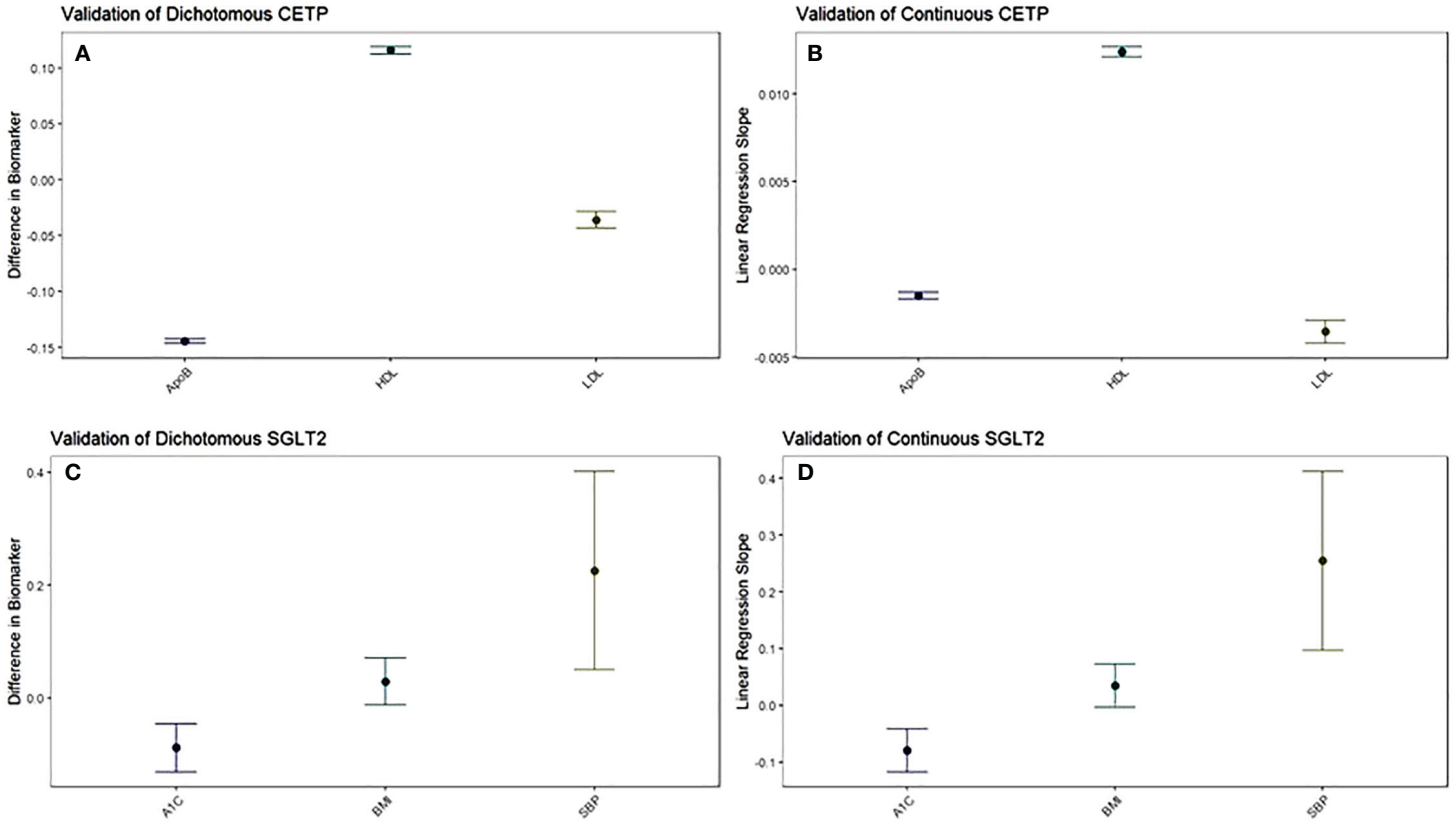
The function of CETP and SGLT2 genetic scores are validated against their target biomarkers. Both dichotomous **(A)** and continuous **(B)** CETP scores are associated with decreased ApoB, increased HDL, and decreased LDL. Both dichotomous **(C)** and continuous **(D)** SGLT2 scores are associated with decreased A1c and increased systolic blood pressure, with increased BMI trending towards significance. For dichotomous scores, point estimates and 95% confidence intervals represent the difference in average biomarker levels between high-CETP and low-CETP groups.

To construct a genetic score that mimics the effects of CETP inhibitors, we build a score of 4 single-nucleotide polymorphisms (SNPs) in the CETP gene region that are strongly correlated with HDL. The genetic score uses all genotyped SNPs in the UK Biobank that were included in a CETP score described in a prior study, Ference et al., 2017 (9, 10). A higher CETP genetic score mimics a greater degree of pharmaceutical CETP inhibition (**Figures 1A, B**).

To construct a genetic score that mimics the effects of SGLT2 inhibitors, we build a score of 2 single-nucleotide polymorphisms (SNPs) in the SGLT2 gene region that are strongly correlated with SGLT2 expression. The genetic score uses all genotyped SNPs in the UK Biobank genotyping information that were included in the SGLT2 score described in Katzmann et al., 2021 (9, 11). A higher SGLT2 genetic score mimics a greater degree of pharmaceutical SGLT2 inhibition (**Figures 1C, D**). For both the CETP and SGLT2 genetic scores, SNPs are filtered for inclusion such that they are all approximately in linkage disequilibrium (LD) (r^2^<0.3) (**Supplementary Tables 1**, **2**).

Our genetic scores are calculated as a linear-additive model, with each effect allele for each included variant weighted based on the effect size of each SNP to its exposure phenotype as calculated by the authors who originally constructed the scores (10, 11) (HDL for CETP SNPs and SGLT2 expression for SGLT2 SNPs). The exact variants, effect alleles, and weights used to construct our genetic scores can be found in **Supplementary Tables 3**, **4**.

### 2.2 Instrumental variable data analysis

**Figure 2:**
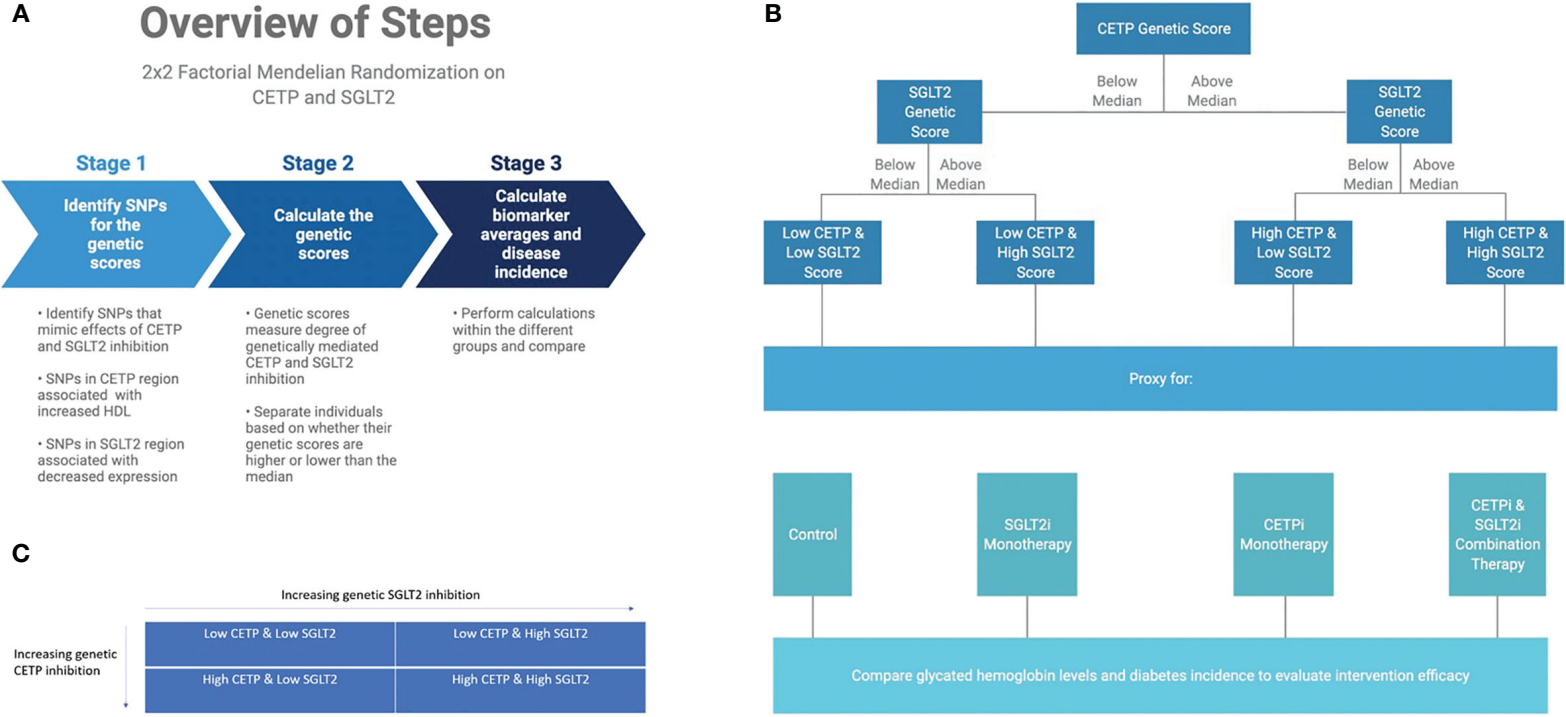
Graphical abstract of methods overview. **(A)** Overview of computational workflow for the 2x2 factorial MR split into 3 steps: identifying SNPs for the genetic scores that mimic effects of CETP and SGLT2 inhibition, calculating the genetic scores, calculating biomarker averages and disease incidence for each of the four groups. **(B)** Description of allocating individuals into one of the four groups: Low CETP and low SGLT2 score, low CETP and high SGLT2 score, high CETP and low SGLT2 score, and high CETP and high SGLT2 score. The same 2x2 factorial MR methods were conducted with each group. **(C)** Once we had our genetic scores and separated the individuals into one of these four groups, we performed a 2x2 factorial MR analysis using UK Biobank with a focus on CETP and SGLT2-relevant datasets.

We perform a 2x2 factorial Mendelian Randomization (MR) analysis using the UK Biobank with a focus on CETP and SGLT2-relevant datasets, as outlined in **Figures 2A, C**. 2x2 factorial MR is a causal inference method that aims to investigate the effect of two different variables, known as exposures, on the same outcome phenotypes. Genetic variants associated with each of the exposure variables are used to build a genetic score that is calculated for each participant in the study. Study participants are then separated into four groups: (1) below the median for both genetic scores (2) above the median for score A but not score B (3) above the median for score B but not score A (4) above the median for both scores. The choice of median as the threshold between groups is meant to create four approximately equally sized groups for comparison. Because participants are separated on the basis of genetic score, and those genetic variants are randomly allocated at birth, 2x2 factorial MR mimics the randomization that allows for causal inference in a randomized controlled trial.

We include all individuals in the UK Biobank that have genotyping information containing all SNPs needed to construct the genetic scores, as well as all biomarker values. Furthermore, only white British individuals are included in the analysis to control for population stratification bias. This is performed because the SGLT2 score from Katzmann et al. (11) is validated only in the white population of the UK Biobank since the eQTLs from which they were constructed were identified in a primarily white population (11, 12). In total, 233,765 individuals fulfilled our study’s inclusion criteria. The final study population is then analyzed with the 2x2 factorial MR methodology detailed in **Figure 2B**.

Individuals are separated into two groups based on whether their CETP genetic score is greater than or less than the median CETP score. In each group, individuals are then separated into two additional groups based on whether their SGLT2 genetic score is greater than or less than the median SGLT2 genetic score. In total, four groups are formed. Then, the mean age, sex, HDL, LDL, TG, ApoB, weight, systolic blood pressure (SBP), glycated hemoglobin, and diabetes incidence rates are recorded for each of the four groups in both the discovery and replication cohorts. Differences in quantitative variables between groups are evaluated using linear regression and differences in diabetes incidence are calculated using Cox proportional hazard models. For proportional hazards modelling, the period from 1995 to 2021 is examined, as those years contain the earliest and latest report of type 2 diabetes diagnosis among our study population.

We conduct analysis controlling for body mass index (BMI) and systolic blood pressure (SBP) using linear regression for glycated hemoglobin and Cox proportional hazards for diabetes, though analysis excluding SBP and BMI as covariates is also conducted. The covariates of BMI and SBP are included in the regression models because the SGLT2 genetic score used to mimic SGLT2 inhibition is associated with increased SBP and BMI, which is the opposite direction of effect that pharmaceutical SGLT2 inhibition has on these variables. BMI and SBP are included as covariates to ensure that the difference in glycated hemoglobin observed between groups is not due to these associations. Diabetes incidence is defined by ICD codes retrieved from the electronic health records database associated with the UK Biobank. All individuals included in this analysis have available health records. Non-additive effects between genetic CETP and SGLT2 scores are detected through linear regressions with CETP, SGLT2, and their product (non-additive term) against glycated hemoglobin and proportional hazards modelling against diabetes incidence. Interaction is detected as a significant p-value in the interaction term of the regression.

### 2.3 Statistical analysis

All analysis is performed in R 4.1.0 (13). All visualization is performed using ggplot2 (14). All hypothesis tests are two-sided and use a statistical significance level of 0.05.

### 2.4 Study/ethics approval

All participants gave written informed consent prior to data collection. UK Biobank has full ethical approval from the NHS National Research Ethics Service (16/NW/0274). All methods were carried out in accordance with relevant guidelines and regulations. UK Biobank data is available to researchers upon request (https://www.ukbiobank.ac.uk/enable-your-research).

## 3 Results

### 3.1 Confirming genetic score function

To ensure that any differences in effects on glycated hemoglobin and diabetes were due to randomization based on our genetic scores, we compared various demographic and biomarker variables between groups (**Table 1**). We observed that among age, biological sex, smoking status, and alcohol status, there were no significant differences between groups. However, we observed statistically significant differences in glycated hemoglobin, diabetes risk, and lipid parameters related to CETP and SGLT2.

**Table 1:** Demographic, biomarker, genetic score, and diabetes data differences between the different groups included in the 2x2 factorial Mendelian randomization design.

	Reference	SGLT2i	CETPi	Combo	P-value
Demographics					
No. of Participants	57 114	45 491	72 986	58 174	
Age	56.84 (8.02)	56.91 (7.98)	56.86 (7.99)	56.82 (8.02)	0.322
Number Male (%)	26 316 (46.1)	20 866 (45.9)	33 597 (46.0)	26 757 (46.0)	0.923
Number Female (%)	30 798 (53.9)	24 625 (54.1)	39 389 (54.0)	31 417 (54.0)	0.923
Smoking					
Never (%)	30 628 (53.6)	24 680 (54.3)	39 797 (54.5)	31 481 (54.1)	0.179
Previous (%)	20 461 (35.8)	16 006 (35.2)	25 556 (35.0)	20 450 (35.2)	0.084
Current (%)	5860 (10.3)	4 632 (10.2)	7 401 (10.1)	6 033 (10.4)	0.605
Prefer not to answer (%)	196 (0.34)	173 (.38)	232 (0.32)	210 (0.36)	0.309
Alcohol					
Daily or almost daily (%)	12 185 (21.3)	9 732 (21.4)	15 477 (21.2)	12 615 (21.7)	0.307
Three or four times a week (%)	13 744 (24.1)	10 953 (24.1)	17 618 (24.1)	13 867 (23.8)	0.722
Once or twice a week (%)	15 069 (26.4)	12 036 (26.5)	19 260 (26.4)	15 279 (26.3)	0.941
One to three times a month (%)	6 385 (11.2)	5 008 (11.0)	8 027 (11.0)	6 506 (11.2)	0.637
Special occasions only (%)	5 960 (10.4)	4 809 (10.6)	7 738 (10.6)	6 065 (10.4)	0.694
Never (%)	3 736 (6.5)	2 926 (6.4)	4 808 (6.6)	3 811 (6.6)	0.784
Prefer not to answer (%)	35 (0.06)	27 (0.06)	58 (0.08)	31 (0.05)	0.269
Medication Use					
Lipid lowering therapy (%)	10 933 (19.1)	8 801 (19.3)	13 264 (18.2)	10 676 (18.4)	9.60E-07
BP lowering therapy (%)	12 574 (22.0)	10 058 (22.1)	15 810 (21.7)	12 498 (21.5)	0.089
Insulin therapy (%)	378 (0.66)	307 (0.68)	450 (0.62)	340 (0.58)	0.214
Lipids					
HDL	1.39 (0.36)	1.40 (0.36)	1.50 (0.39)	1.50 (0.39)	2.00E-16
LDL	3.60 (0.86)	3.60 (0.86)	3.57 (0.85)	3.56 (0.85)	2.00E-16
TG	1.76 (0.99)	1.76 (1.00)	1.72 (0.97)	1.72 (0.97)	2.00E-16
ApoB	1.04 (0.24)	1.04 (0.24)	1.03 (0.24)	1.03 (0.24)	2.00E-16
Non-lipid biomarkers					
Glycated hemoglobin	35.58 (4.77)	35.52 (4.76)	35.53 (4.72)	35.44 (4.68)	9.06E-06
Body mass index	27.36 (4.70)	27.42 (4.70)	27.36 (4.70)	27.33 (4.70)	0.0223
Systolic blood pressure	140.00 (19.59)	140.37 (19.72)	139.97 (19.61)	140.10 (19.62)	0.0042
Genetic scores					
CETP score	13.93 (3.50)	13.90 (3.53)	22.41 (2.92)	22.44 (2.92)	2.00E-16
SGLT2 score	0.56 (0.27)	1.47 (0.15)	0.56 (0.27)	1.47 (0.15)	2.00E-16
**Diabetes cases (%)**	3806 (6.67)	3014 (6.63)	4753 (6.51)	3637 (6.25)	0.029

All lipid measurements have units of mmol/L and glycated hemoglobin is reported in units of mmol/mol. Blood pressure is reported in units of mmHg. Genetic scores have arbitrary units. P-values are derived from significance testing for differences between each of the groups. ANOVA is used for quantitative variables while chi-squared tests are used for categorical variables. Statistically significant differences between groups are highlighted in red.

Pharmaceutical CETP inhibition is known to increase HDL and decrease LDL along with ApoB (15–19). Thus, we validated whether our CETP genetic score behaved similarly to pharmaceutical CETP inhibition by seeing if elevated CETP genetic scores, corresponding to more CETP inhibition, exhibited these same relationships. This validation was performed through a dichotomous CETP score, where participants are split into high and low groups and differences in lipid biomarkers are observed, as well as through a continuous CETP score, where the score itself is regressed against HDL, LDL, and ApoB. We find both scores are strongly associated with increased HDL, decreased LDL, and decreased ApoB with p-values less than 2x10^-16^ for all comparisons (**Figures 1A, B**; **Supplementary Table 5**). Based on these results, our CETP score is likely a reasonable proxy for pharmaceutical CETP inhibition.

Pharmaceutical SGLT2 inhibition decreases glycated hemoglobin, systolic blood pressure, and body-mass index, so we validate the function of our SGLT2 genetic score based on associations with these biomarkers (20–23). We perform the same analysis with dichotomous and continuous scores as detailed above to validate our CETP genetic score, finding that our SGLT2 score is associated with decreased glycated hemoglobin (dichotomized: p=0.0000426; continuous: p=0.000044), but increased systolic blood pressure (dichotomized: p=0.0116; continuous: p=0.00149) with increased BMI trending towards significance (dichotomized: p=0.172; continuous: p=0.0708) (**Figures 1C, D**; **Supplementary Table 5**). Based on these results, we conclude that our SGLT2 score functions as expected with respect to glycated hemoglobin, but not with respect to SBP and BMI. Since the primary mechanism of action of SGLT2 inhibition is on glycated hemoglobin with blood pressure and BMI effects coming secondary to that, we believe our SGLT2 score is likely a reasonable proxy for pharmaceutical SGLT2 inhibition. However, we will control for the possible confounding of the SBP and BMI associations by including them as covariates in all aspects of the following 2x2 factorial Mendelian Randomization analysis. For each comparison between groups there will be two statistical tests performed: one without SBP and BMI as covariates and one including SBP and BMI as covariates.

### 3.2 Comparison of glycated hemoglobin between groups

**Figure 3:**
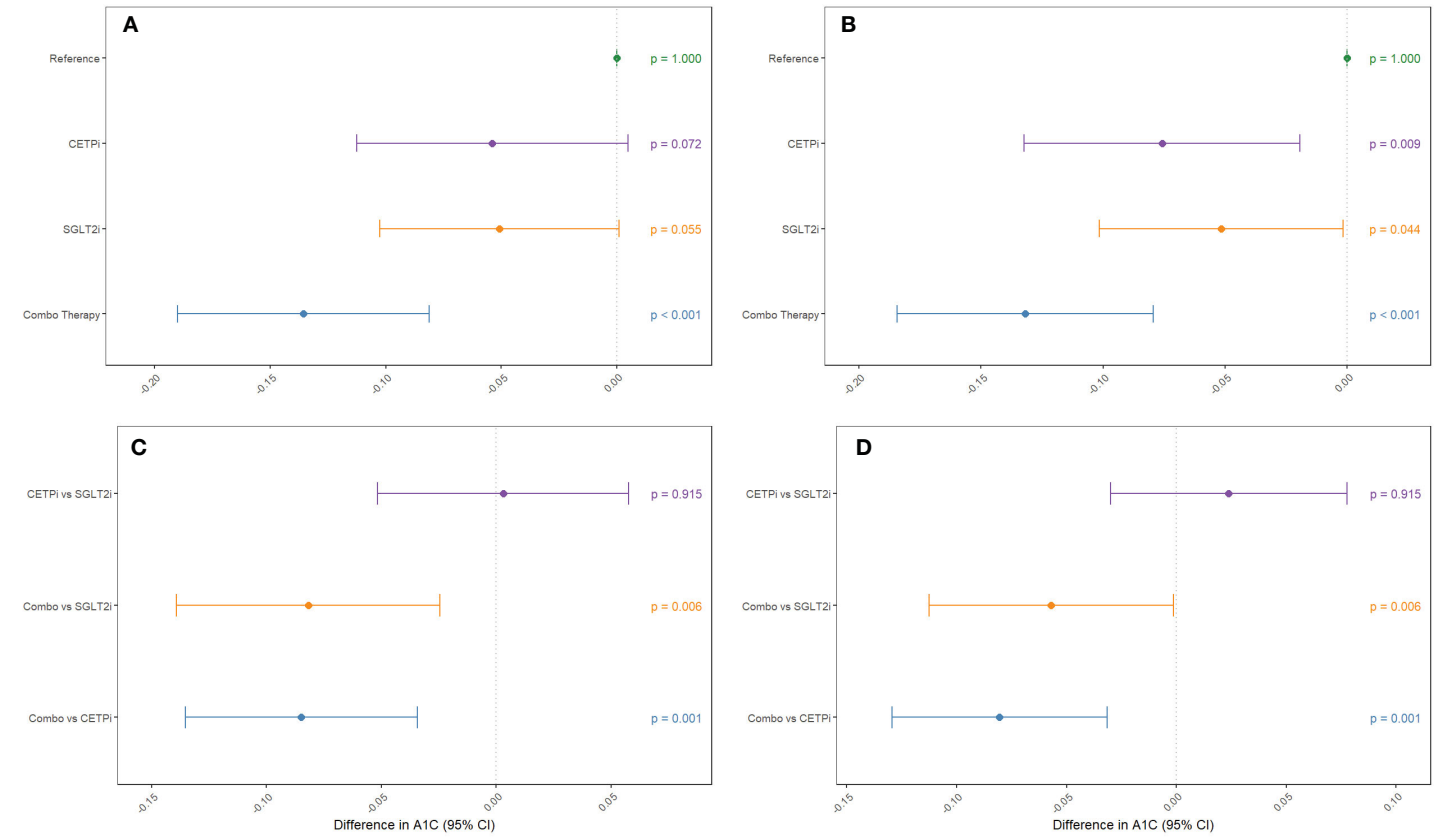
Groups mimicking CETP inhibition, SGLT2 inhibition, and inhibition of both CETP and SGLT2 are compared against control to examine their effect on decreasing glycated hemoglobin levels. This comparison is performed **(A)** without SBP and BMI as covariates and **(B)** with SBP and BMI as covariates. Non-control groups are compared **(C)** without SBP and BMI as covariates and **(D)** with SBP and BMI as covariates.

After confirming the function of our scores and identifying SBP and BMI as possible confounders to control for, we proceed with our 2x2 factorial Mendelian Randomization analysis and split our cohort into four groups corresponding to control, SGLT2 inhibition only (SGLT2i), CETP inhibition only (CETPi), and both SGLT2 and CETP inhibition (combo therapy). We compare the levels of glycated hemoglobin between members of each group and the reference group (low CETP and low SGLT2 score). We find that both SGLT2i (p=0.0719) and CETPi (p=0.0549) are associated with decreased glycated hemoglobin relative to control, and this signal is strengthened to statistical significance (SGLT2i: p=0.0088; CETPi: p=0.044) when SBP and BMI are included as covariates (**Figures 3A, B**; **Supplementary Table 6**). Furthermore, when the non-control groups are compared against each other, no significant difference is found between the CETPi and SGLT2i groups (p=0.915), but the combo therapy group has significantly lower glycated hemoglobin levels than the SGLT2i (p=0.00558) and CETPi (p=0.00118) groups; this conclusion is recapitulated when SBP and BMI are included as covariates (CETPi vs SGLT2i: p=0.384; Combo vs SGLT2i: p=0.0459; Combo vs CETPi: p=0.00146) (**Figures 3C, D**; **Supplementary Table 6**). Non-additive effects between genetic CETP and SGLT2 inhibition on glycated hemoglobin are not detected, as the interaction term is not significantly associated with glycated hemoglobin (Estimate: 0.0000913; 95% CI: -0.0007 to 0.0007; p-value=0.98) (**Supplementary 7**).

### 3.3 Comparison of diabetes risk between groups

**Figure 4:**
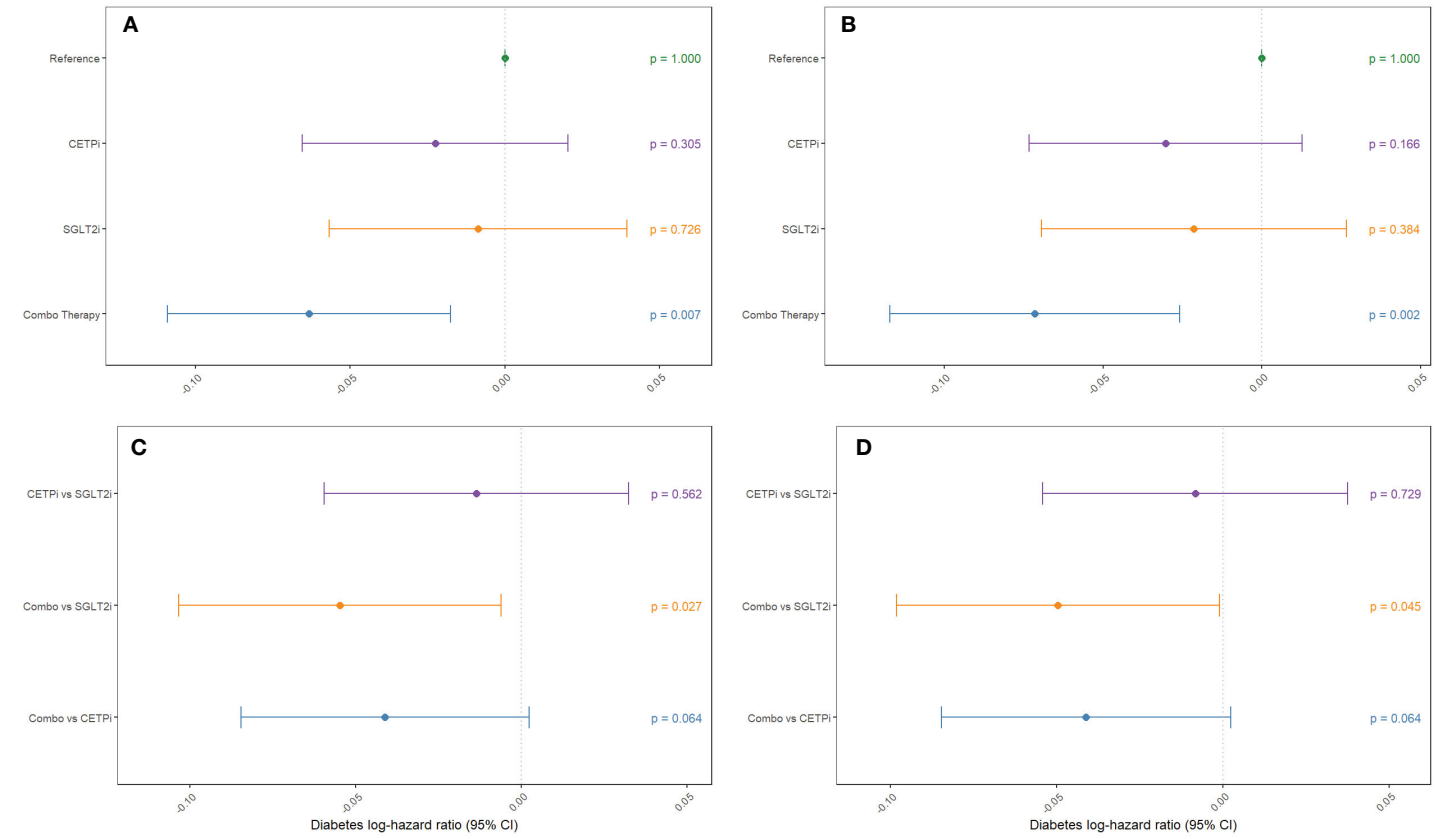
Groups mimicking CETP inhibition, SGLT2 inhibition, and inhibition of both CETP and SGLT2 are compared against control to examine their effect on decreasing diabetes incidence. This comparison is performed **(A)** without SBP and BMI as covariates and **(B)** with SBP and BMI as covariates. Non-control groups are compared **(C)** without SBP and BMI as covariates and **(D)** with SBP and BMI as covariates.

To determine whether joint CETP and SGLT2 inhibition has an impact on diabetes risk within each of our groups, we leverage the same analytical framework as detailed above for glycated hemoglobin but use Cox proportional hazards modelling instead of linear regression. We find that neither CETPi nor SGLT2i are associated with decreased diabetes risk relative to control (CETPi: p=0.305; SGLT2i: p=0.726), but combo therapy is associated with significantly decreased diabetes risk relative to control (p=0.00666) (**Figure 4A**; **Supplementary Table 8**). These findings are unchanged if SBP and BMI are included as covariates (CETPi: p=0.166; SGLT2i: p=0.384; Combo: p=0.00222) (**Figure 4B**; **Supplementary Table 8**). Comparison between groups suggests that SGLT2i and CETPi do not have significantly different incidence of diabetes (p=0.562), though the combo therapy group is significantly associated with decreased diabetes incidence relative to SGLT2i (p=0.0273) and is trending towards significance relative to CETPi (p=0.0635) (**Figure 4C**; **Supplementary Table 8**). Replication of these findings is achieved when SBP and BMI are included as covariates (CETPi vs SGLT2i: p=0.729; Combo vs SGLT2i: p=0.0454; Combo vs CETPi: p=0.0635) (**Figure 4D**; **Supplementary Table 8**). Non-additive effects between genetic CETP and SGLT2 inhibition on diabetes risk are not detected, as the interaction term is not significantly associated with diabetes risk (Estimate: -0.00233; 95% CI: -0.008 to 0.004; p-value=0.454) (**Supplementary 7**). A summary of all evaluated quantities between different groups in the 2x2 factorial framework is presented in **Table 1**.

## 4 Discussion

### 4.1 Genetic evidence for efficacy of CETP and SGLT2 inhibitor combination therapy for increasing glycemic control

In this study, we have used a 2x2 factorial Mendelian Randomization framework to investigate the effects of SGLT2i and CETPi combination therapy on glycated hemoglobin levels and diabetes risk. We find that combination therapy is associated with decreased glycated hemoglobin levels compared to SGLT2i, CETPi, and control, with this conclusion robust against the inclusion of potential confounders (SBP and BMI) as covariates. The combination therapy group also had significantly lower diabetes incidence compared to both control and SGLT2i, and was trending towards significance for CETPi. We detected no evidence of interactions between genetic CETP and SGLT2 inhibition on either glycated hemoglobin or diabetes risk. Taken together, these results constitute genetics-driven evidence suggesting that combination therapy with CETP and SGLT2 inhibitors confers improved protection against hyperglycemia and diabetes risk compared to SGLT2 inhibitors alone. Furthermore, the lack of interaction effect suggests that genetic CETP or SGLT2 inhibition does not attenuate the effect of the other on glycated hemoglobin or diabetes risk.

### 4.2 CETP and SGLT2 inhibitor combination therapy is a potential oral therapeutic alternative to insulin

It is important to note that since CETP and SGLT2 inhibitors are both oral drugs, their combination therapy represents an oral therapeutic strategy for treatment-resistant diabetes prior to use of injectable drugs such as insulin or glucagon-like peptide 1 (GLP1) receptor agonists (24). The ease of taking oral therapy over injectable drugs could lead to higher patient compliance to drive better patient outcomes as well as lower metabolic disease morbidity. Indeed, previous investigations of medication adherence in type 2 diabetes estimate adherence of oral hyperglycemia agents to be between 38% and 93%, generally higher than that of injectable hyperglycemia agents, which are between 38% and 61% (25–29).

### 4.3 2x2 factorial Mendelian randomization is a promising screening method for repurposing opportunities and combination therapies

The cost of bringing a new drug to market is exceptionally high, costing over $1 billion with estimates ranging as high as $2.6 billion (30, 31). Beyond heavy monetary investment, developing medicine to treat a disease from scratch requires a time investment of over a decade (31, 32). It is important to maximize the patient-benefit derived from this investment, and one method of doing so is identifying repurposing opportunities for existing drugs in related disease phenotypes. Critically, since CETP inhibitors such as obicetrapib are currently in phase 3 clinical trials to treat dyslipidemia and coronary artery disease, its clean safety and toxicity profile makes it a good repurposing candidate for metabolic disease and combination therapies. Our analysis not only provides genetics-driven support for repurposing CETP inhibitors to metabolic disease in the form of a CETP and SGLT2 inhibitor combination therapy, but also highlights the power of 2x2 factorial Mendelian Randomization as a framework through which combination therapies across disease verticals can be systematically discovered and mined for repurposing opportunities supported by human genetic validation. Previous 2x2 factorial MR studies have identified combination therapies primarily within cardiovascular disease between PCSK9 and CETP inhibition, NPC1L1 and HMGCR inhibition, and IL-6 and PCSK9/CETP/NPC1L1 inhibition (8, 33, 34). However, to the best of our knowledge, this study is the first to identify 2x2 factorial MR as a way to identify phase 3 cardiovascular drugs that can be repurposed for metabolic disease in a combination therapy. This conclusion is published under World Intellectual Property Organization patent WO2023129595A1 (35).

### 4.4 Limitations

Despite its powerful conclusion, our study is not without limitations. The primary limitation of our analysis is the fact that we did not actually administer pharmacologic SGLT2 or CETP inhibitors to investigate the effect of either monotherapy. Rather, we analyzed the effect of lifetime decreased SGLT2 or CETP function caused by genetic variation, which could differ from the shorter acting effects of pharmaceutical therapies. Since our approach is grounded in genetics, it also does not account for the potential off-target effects of small molecule inhibitors of either SGLT2 or CETP, which can only be ascertained through a randomized control trial. However, our analysis does provide strong genetics-driven evidence grounded in a proven causal inference framework that can be used to inform future clinical trials of this combination therapy.

Furthermore, the actual instruments may have pleiotropic effects on glycated hemoglobin or lipid parameters independent of their effect on SGLT2 or CETP function, though we have attempted to minimize this potential source of confounding by using previously validated genetic scores from the literature and adjusting for biomarkers as covariates.

In our SGLT2 score, we consider the pleiotropic effects of variant rs3116150, where the allele linked to reduced SGLT2 expression also correlates with higher TGFB1I1 expression. Given that TGFB1I1 expression shows no significant relationship with glycated hemoglobin or diabetes in prior studies, its impact is likely not influencing our phenotypes of interest. However, TGFB1I1 expression is associated with higher blood pressure (36), which is a potential explanation for the unexpected link between our SGLT2 score and elevated systolic blood pressure, diverging from the blood pressure reduction seen with SGLT2 inhibitor drugs.

Our study leverages the SGLT2 score developed by Katzmann et al., 2021, which uses variants tied to SGLT2 expression in non-renal tissues, despite SGLT2’s primary action in the kidneys. However, the lack of association with SGLT2 expression in the kidneys is potentially due to the low sample size of kidneys in Genotype-Tissue Expression Project, of which there are only 80, compared to >500 samples of other tissues. Despite this, our validation confirms the score’s link to lower glycated hemoglobin levels. Furthermore, Katzmann et al. identified associations of the score with reduced heart failure and uric acid levels. All these effects are consistent with those of pharmaceutical SGLT2 inhibitors. While our score is linked to higher systolic blood pressure, our core conclusions remain robust even when blood pressure is included as a covariate in our models.

Lastly, our analysis demonstrates the effectiveness joint CETP and SGLT2 inhibition in a white population within the UK Biobank, but the strength and generalizability of our analysis would be increased by a more ethnically diverse data cohort such as the NIH All of Us Research Program (37). This endeavor would be facilitated by the construction of genetic scores of CETP and SGLT2 variants that are more translatable across different populations.

### 4.5 Conclusions

Our results suggest that joint genetic inhibition of CETP and SGLT2 leads to decreased glycated hemoglobin and decreased risk of diabetes. This highlights a potential drug repurposing opportunity, where CETP inhibitors, which are currently in clinical trials for cardiovascular disease, can be combined with SGLT2 inhibitors to create a combination therapy to treat type 2 diabetes. Importantly, this would represent an additional oral therapeutic alternative to insulin for diabetic patients, potentially leading to increased medication adherence and improved health outcomes. Further research may extend our genetics-driven conclusion through clinical trials involving CETP and SGLT2 combination therapy.

## Data availability statement

Publicly available datasets were analyzed in this study. This data can be found here: https://www.ukbiobank.ac.uk/enable-your-research.

## Ethics statement

Ethical approval was not required for the study involving humans in accordance with the local legislation and institutional requirements. Written informed consent to participate in this study was not required from the participants or the participants’ legal guardians/next of kin in accordance with the national legislation and the institutional requirements.

## Author contributions

BK: Writing – review & editing, Writing – original draft, Visualization, Validation, Supervision, Software, Resources, Project administration, Methodology, Investigation, Funding acquisition, Formal Analysis, Data curation, Conceptualization. PS: Writing – review & editing, Writing – original draft, Visualization, Validation, Software, Methodology, Investigation, Formal Analysis, Data curation. ZM: Validation, Writing – review & editing. MD: Writing – original draft, Supervision, Conceptualization, Writing – review & editing. JK: Writing – review & editing. MD: Writing – review & editing, Supervision, Project administration.

## References

[1] Hirode G Wong RJ. Trends in the prevalence of metabolic syndrome in the United States, 2011-2016. JAMA. (2020) 323:2526. doi: 10.1001/jama.2020.4501 32573660 PMC7312413

[2] Shyr ZA Yan Z Ustione A Egan EM Remedi MS. SGLT2 inhibitors therapy protects glucotoxicity-induced β-cell failure in a mouse model of human KATP-induced diabetes through mitigation of oxidative and ER stress. PloS One. (2022) 17:e0258054. doi: 10.1371/journal.pone.0258054 35180212 PMC8856523

[3] Heerspink HJL Perkins BA Fitchett DH Husain M Cherney DZI. Sodium glucose cotransporter 2 inhibitors in the treatment of diabetes mellitus: cardiovascular and kidney effects, potential mechanisms, and clinical applications. Circulation. (2016) 134:752–72. doi: 10.1161/CIRCULATIONAHA.116.021887 27470878

[4] DeFronzo RA. From the triumvirate to the ominous octet: a new paradigm for the treatment of type 2 diabetes mellitus. Diabetes. (2009) 58:773–95. doi: 10.2337/db09-9028 PMC2661582 19336687

[5] Guo W Gong Y Fu Z Fu J Sun Y Ju X. The effect of cholesteryl ester transfer protein on pancreatic beta cell dysfunction in mice. Nutr Metab (Lond). (2016) 13:21. doi: 10.1186/s12986-016-0082-1 26973702 PMC4788865

[6] Fryirs M Barter P Appavoo M Tuch B Tabet F Heather A. Effects of high-density lipoproteins on pancreatic β-cell insulin secretion. ATVB. (2010) 30:1642–8. doi: 10.1161/ATVBAHA.110.207373 20466975

[7] Masson W Lobo M Siniawski D Huerin M Molinero G Valero R. Therapy with cholesteryl ester transfer protein (Cetp) inhibitors and diabetes risk. Diabetes Metab. (2018) 44:508–13. doi: 10.1016/j.diabet.2018.02.005 29523487

[8] Ference BA Majeed F Penumetcha R Flack JM Brook RD. Effect of naturally random allocation to lower low-density lipoprotein cholesterol on the risk of coronary heart disease mediated by polymorphisms in npc1l1, hmgcr, or both. J Am Coll Cardiol. (2015) 65:1552–61. doi: 10.1016/j.jacc.2015.02.020 PMC6101243 25770315

[9] Bycroft C Freeman C Petkova D Band G Elliot L Sharp K. The UK Biobank resource with deep phenotyping and genomic data. Nature. (2018) 562:203–9. doi: 10.1038/s41586-018-0579-z PMC6786975 30305743

[10] Ference BA Kastelein JJP Ginsberg HN Chapman M Nicholls S Ray K. Association of genetic variants related to cetp inhibitors and statins with lipoprotein levels and cardiovascular risk. JAMA. (2017) 318:947–56. doi: 10.1001/jama.2017.11467 PMC5710502 28846118

[11] Katzmann JL Mason AM März W Kleber M Niessner A Bluher M. Genetic variation in sodium-glucose cotransporter 2 and heart failure. Clin Pharmacol Ther. (2021) 110:149–58. doi: 10.1002/cpt.2153 33405238

[12] Lonsdale J Thomas J Salvatore M Philips R Lo E Shad S. The genotype-tissue expression (Gtex) project. Nat Genet. (2013) 45:580–5. doi: 10.1038/ng.2653 PMC4010069 23715323

[13] R: the r project for statistical computing. Available online at: https://www.r-project.org/ (Accessed April 30, 2023).

[14] Wickham H. Ggplot2: Elegant Graphics for Data Analysis. 2nd ed. Springer-Verlag New York: Springer International Publishing (2016). https://ggplot2.tidyverse.org.

[15] Tall AR Rader DJ. Trials and tribulations of cetp inhibitors. Circ Res. (2018) 122:106–12. doi: 10.1161/CIRCRESAHA.117.311978 PMC5756107 29018035

[16] Holmes MV Smith GD. Revealing the effect of CETP inhibition in cardiovascular disease. Nat Rev Cardiol. (2017) 14:635–6. doi: 10.1038/nrcardio.2017.156 PMC5644574 28980665

[17] Nicholls SJ Ditmarsch M Kastelein JJ Rigby S Kling D Curcio D. Lipid lowering effects of the CETP inhibitor obicetrapib in combination with high-intensity statins: a randomized phase 2 trial. Nat Med. (2022) 28:1672–8. doi: 10.1038/s41591-022-01936-7 35953719

[18] Nicholls SJ Brewer HB Kastelein JJP Krueger K Wang M Shao M. Effects of the cetp inhibitor evacetrapib administered as monotherapy or in combination with statins on hdl and ldl cholesterol: a randomized controlled trial. JAMA. (2011) 306:2099–109. doi: 10.1001/jama.2011.1649 22089718

[19] Nicholls SJ. Cetp-inhibition and hdl-cholesterol: a story of cv risk or cv benefit, or both. Clin Pharmacol Ther. (2018) 104:297–300. doi: 10.1002/cpt.1118 29901215

[20] Kawasoe S Maruguchi Y Kajiya S Uenomachi H Miyata M Kawasoe M. Mechanism of the blood pressure-lowering effect of sodium-glucose cotransporter 2 inhibitors in obese patients with type 2 diabetes. BMC Pharmacol Toxicol. (2017) 18:23. doi: 10.1186/s40360-017-0125-x 28391776 PMC5385592

[21] Cho YK Kim YJ Jung CH. Effect of sodium-glucose cotransporter 2 inhibitors on weight reduction in overweight and obese populations without diabetes: a systematic review and a meta-analysis. J Obes Metab Syndr. (2021) 30:336–44. doi: 10.7570/jomes21061 PMC8735829 34897070

[22] Teo YH Teo YN Syn NL Kow C Yoong C Tan B. Effects of sodium/glucose cotransporter 2 (Sglt2) inhibitors on cardiovascular and metabolic outcomes in patients without diabetes mellitus: a systematic review and meta-analysis of randomized-controlled trials. JAHA. (2021) 10:e019463. doi: 10.1161/JAHA.120.019463 33625242 PMC8174267

[23] Adamson C Kondo T Jhund PS Boer R Honorio J Claggett B. Dapagliflozin for heart failure according to body mass index: the DELIVER trial. Eur Heart J. (2022) 43:4406–17. doi: 10.1093/eurheartj/ehac481 PMC9622300 36029309

[24] Armitage J Holmes MV Preiss D. Cholesteryl ester transfer protein inhibition for preventing cardiovascular events. J Am Coll Cardiol. (2019) 73:477–87. doi: 10.1016/j.jacc.2018.10.072 PMC6354546 30704580

[25] Alatorre C Fernández Landó L Yu M Brown K Montejano L Juneau P. Treatment patterns in patients with type 2 diabetes mellitus treated with glucagon-like peptide-1 receptor agonists: Higher adherence and persistence with dulaglutide compared with once-weekly exenatide and liraglutide: ALATORRE. Diabetes Obes Metab. (2017) 19:953–61. doi: 10.1111/dom.12902 PMC5485056 28181725

[26] Krass I Schieback P Dhippayom T. Adherence to diabetes medication: a systematic review. Diabetes Med. (2015) 32:725–37. doi: 10.1111/dme.12651 25440507

[27] Mody R Huang Q Yu M Zhao R Patel H Grabner M. Adherence, persistence, glycaemic control and costs among patients with type 2 diabetes initiating dulaglutide compared with liraglutide or exenatide once weekly at 12-month follow-up in a real-world setting in the United States. Diabetes Obes Metab. (2019) 21:920–9. doi: 10.1111/dom.13603 PMC6590811 30520248

[28] Mody R Manjelievskaia J Marchlewicz E Zimmerman N Irwin D Yu M. Greater adherence and persistence with injectable dulaglutide compared with injectable semaglutide at 1-year follow-up: data from us clinical practice. Clin Ther. (2022) 44:537–54. doi: 10.1016/j.clinthera.2022.01.017 35264311

[29] Yu M Xie J Fernandez Lando L Kabul S Swindle RW. Liraglutide versus exenatide once weekly: persistence, adherence, and early discontinuation. Clin Ther. (2016) 38:149–60. doi: 10.1016/j.clinthera.2015.11.017 26706658

[30] Wouters OJ McKee M Luyten J. Estimated research and development investment needed to bring a new medicine to market, 2009-2018. JAMA. (2020) 323:844. doi: 10.1001/jama.2020.1166 32125404 PMC7054832

[31] Mohs RC Greig NH. Drug discovery and development: Role of basic biological research. Alzheimer’s Dementia: Trans Res Clin Intervent. (2017) 3:651–7. doi: 10.1016/j.trci.2017.10.005 PMC5725284 29255791

[32] Tamimi NAM Ellis P. Drug development: from concept to marketing! Nephron Clin Pract. (2009) 113:c125–31. doi: 10.1159/000232592 19729922

[33] Cupido A Reeskamp L Hingorani A Finan C Asselbergs F Hovingh G. Joint genetic inhibition of pcsk9 and cetp and the association with coronary artery disease: a factorial mendelian randomization study. JAMA Cardiol. (2022) 7:955. doi: 10.1001/jamacardio.2022.2333 35921096 PMC9350849

[34] Georgakis MK Malik R Burgess S Dichgans M. Additive effects of genetic interleukin-6 signaling downregulation and low-density lipoprotein cholesterol lowering on cardiovascular disease: a 2×2 factorial mendelian randomization analysis. JAHA. (2022) 11:e023277. doi: 10.1161/JAHA.121.023277 34927447 PMC9075213

[35] Davidson M Cui S, Inventors; NewAmsterdam Pharma BV, applicant. Obicetrapib, SGLT2 inhibitor combination. World Intellectual Property Organization patent WO2023129595A1. (2023).

[36] Fedoseeva L Klimov L Ershov N Alexandrovich Y Efimov V Markel A. Molecular determinants of the adrenal gland functioning related to stress-sensitive hypertension in ISIAH rats. BMC Genomics. (2016) 17:989. doi: 10.1186/s12864-016-3354-2 28105924 PMC5249038

[37] The All of Us Research Program Investigators. The “all of us” research program. N Engl J Med. (2019) 381:668–76. doi: 10.1056/NEJMsr1809937 PMC8291101 31412182

